# Obesity‐associated glomerular inflammation increases albuminuria without renal histological changes

**DOI:** 10.1002/2211-5463.12400

**Published:** 2018-02-26

**Authors:** Akira Mima, Toshinori Yasuzawa, George L. King, Shigeru Ueshima

**Affiliations:** ^1^ Department of Nephrology Kindai University Faculty of Medicine Kindai University Nara Hospital Nara Japan; ^2^ Department of Food Science and Nutrition Faculty of Agriculture Kindai University Nara Japan; ^3^ Research Division Joslin Diabetes Center Harvard Medical School Boston MA USA; ^4^ Department of Applied Biological Chemistry Graduate School of Agriculture Kindai University Nara Japan; ^5^ Antiaging Center Kindai University Osaka Japan

**Keywords:** albuminuria, CKD, Inflammation, NF‐κB, obesity

## Abstract

Obesity is one of risk factors for chronic kidney disease (CKD), but the precise mechanism involved is unclear. This study characterizes the effect of obesity‐induced glomerular inflammation, oxidative stress, and albuminuria in obese rats. Glomerular samples were collected from fatty (ZF) and lean (ZL) Zucker rats. After 2 months of feeding, body weight and albuminuria were significantly increased in ZF rats when compared to ZL rats. Expression of the inflammatory markers TNF‐α and CCR2 was significantly increased in the glomeruli of ZF rats. However, expression of IL‐6 mRNA was not increased. Analysis of renal pathology showed no glomerular expansion. As inflammatory and oxidative stress markers are associated with NF‐κB, we evaluated whether NF‐κB activation was increased in the glomeruli of mice on a high‐fat diet. Immunohistochemistry showed increased NF‐κB activation in the glomeruli when transgenic mice overexpressing an NF‐κB‐dependent enhanced green fluorescent protein were fed with a high‐fat diet. These results suggest that obesity of only 2 months duration can cause albuminuria, due to increased inflammation or oxidative stress, but may not be long enough to develop renal pathological changes.

**Abbreviations**
CKD: chronic kidney disease
IRS: insulin receptor substrate
NF‐κB: nuclear factor κ B
SHP‐1: Src homology‐domain‐containing phosphatase‐1
VEGF: vascular endothelial growth factor
ZF: fatty Zucker rats
ZL: lean Zucker rats

Obesity is one of the common causes of chronic kidney disease (CKD), independent of glycemic control 1. Recent studies suggest that body mass index is associated with the incidence of CKD 2. We have shown that obesity‐induced abnormal metabolites may play a significant role in increasing vascular endothelial growth factor (VEGF) and subsequently the development of CKD 3. Furthermore, insulin resistance observed in obesity has been associated with cardiovascular disease 4. These results suggest that obesity‐induced abnormal metabolites may accelerate the development of CKD.

Albuminuria is an early abnormal feature of CKD and has been recognized as a marker of systemic endothelial dysfunction 5. Thus, albuminuria could reflect worsening renal function, cardiovascular disease, and increased risk of mortality 6. Data from the Prevention of Renal and Vascular End Stage Disease (PREVEND) study clearly show that increased albuminuria not only follows overt diabetic kidney disease, but is also a marker of the progression of diabetes 7. We have shown that increases in inflammation and oxidative stress are recognized in the glomerular endothelial cells in both diabetes and obesity‐induced insulin resistance states 3. Recent studies suggest that inflammatory markers are closely related to endothelial dysfunction, which has been shown to indicate the development of diabetes 8, 9, 10.

This study characterized the mechanism of albuminuria caused by inflammation and oxidative stress in the glomeruli of obese and insulin‐resistant rats.

## Research design and methods

### Animal studies

All animal protocols were approved by the Kindai University and Joslin Diabetes Center's Animal Care Committee in accordance with the National Institutes of Health guidelines. We used age‐matched male ZF, lean ZL rats, and C57BL/6J mice (Shimizu, Kyoto, Japan). To determine nuclear factor κΒ (NF‐κΒ) activation in the glomeruli, we used NF‐κΒ‐dependent enhanced green fluorescent protein (GFP) transgenic mice (cis‐NF‐κΒ^EGFP^) 11. These mice were produced as described previously and kindly provided by Steve Shoelson and Jongsoon Lee at the Joslin Diabetes Center. Obesity and insulin‐resistant states were induced in 8‐week‐C57BL/6J and 8‐week‐cis‐NF‐κΒ^EGFP^ mice by feeding them a high‐fat diet (45% and 42% from fat; Shimizu, Kyoto, Japan and Harlan Tekland, Indianapolis, IN, USA, respectively) or a normal diet for 2 months. Eight‐week‐ cis‐NF‐κΒ^EGFP^ mice were the same group as published in our previous study 11.

### Isolation of glomeruli

Rat glomeruli were isolated from the renal cortex by the sieving method as described previously 3.

### DNA fragmentation analysis

DNA fragmentation was measured by quantitation of cytosolic oligonucleosome‐bound DNA using an ELISA, according to the manufacturer's instructions (Roche Diagnostics, Indianapolis, IN, USA).

### Measurement of urinary albumin

Albuminuria was measured by Nephrat or Albuwell (Exocell Inc., Philadelphia, PA, USA) using 24‐h urine collection samples from animals housed in individual metabolic cages.

### Serum triglyceride, serum total cholesterol, and plasma insulin

Serum triglyceride and serum total cholesterol were measured by LabAssay Triglyceride (Wako Chemicals, Richmond, VA, USA) and by LabAssay Cholesterol (Wako Chemicals, Richmond, VA, USA), respectively. Plasma insulin was measured by Ultra Sensitive Rat Insulin ELISA Kit (Morinaga Institute of Biological Science, Yokohama, Japan).

### Real‐time PCR analysis

Total RNA was isolated from the glomeruli using an RNAeasy microcolumn with DNase treatment (Qiagen, Valencia, CA, USA). Quantification of RNA was performed with the NanoDrop ND‐1000 spectrophotometer (Thermo Scientific, Wilmington, DE, USA). cDNA was synthesized using Superscript III reverse transcriptase (Invitrogen, Carlsbad, CA, USA). mRNA expression in the glomeruli was evaluated by a SYBR green procedure (Applied Biosystems, Foster City, CA, USA). Amplification and detection were performed using the Step One Plus system (Applied Biosystems). Expression levels were normalized to levels of GAPDH. PCR primers were as follows: TNF‐α AAATGGGCTCCCTCTCATCAGTTC, TCTGCTTGGTTTGCTACGAC; IL‐6 TCCTACCCCAACTTCCAATGCTC, TTGGATGGTCTTGGTCCTTAGCC; CCR2 CTTGTGGCCCTTATTTTCCA, GAATTCCTGGAAGGTGGTCA; and GAPDH GTATTGGGCGCCTGGTCACC, CGCTCCTGGAAGATGGTGATGG.

### Histological study

Kidney samples for light microscopy analysis were fixed in 4% paraformaldehyde phosphate buffer. Kidney sections (2 μm) were stained with periodic acid–Schiff. Glomeruli were digitally photographed, and the images were imported into imagej software (National Institutes of Health, Bethesda, MD, USA; https://imagej.nih.gov/ij/) and analyzed morphometrically. Dissected glomeruli from obese and control cis‐NF‐κΒ^EGFP^ mice were fixed in acetone and observed by digital fluorescence microscopy.

### Data analysis

Data are expressed as mean ± SD. Comparisons among more than two groups were performed by one‐way ANOVA, followed by post hoc analysis with paired or unpaired *t*‐test to evaluate statistical significance. All analyses were performed using StatView (SAS Institute, Cary, CA, USA). Statistical significance was defined as *P* < 0.05.

## Results

### Physiological characteristics of experimental groups

**Figure 1:**
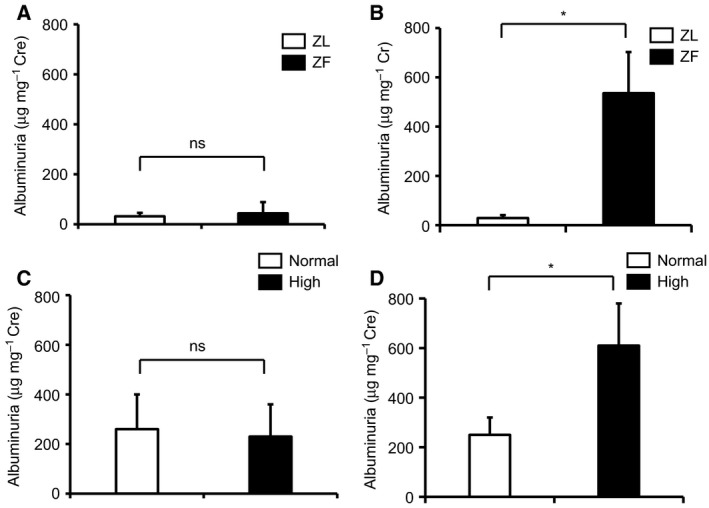
Albuminuria in experimental groups. (A) Albuminuria in ZL and ZF rats at the start of experiment. (B) Albuminuria in ZL and ZF rats at 2 months. (C) Albuminuria in mice at the start of experiment. (D) Albuminuria in mice fed with high‐fat chow after 5 months. *P < 0.05. ns, not significant. These data are expressed as means ± SD. ZL, Zucker lean rats; ZF, Zucker fatty rats.

Body weight was significantly increased in ZF rats by 1.6 ± 0.1‐fold when compared to ZL rats. Like ZF rats, mice fed with high‐fat chow showed increases in body weight when compared to mice fed with normal chow (Tables 1 and 2, *P* < 0.05). Plasma triglyceride and cholesterol levels in ZF rats were elevated by 7.3 ± 4.6‐fold and 1.3 ± 0.6‐fold, respectively, compared to ZL rats (*P* < 0.05). However, there were no significant statistical differences in the levels of plasma insulin (Table 3). Initially, there were no significant differences in albuminuria between ZF and ZL rats (Fig. 1A). However, after 2 months of feeding, albuminuria was significantly increased in ZF rats by 30 ± 20‐fold when compared to ZL rats (Fig. 1B, *P* < 0.05). Like rat experiments, there are no significant differences in albuminuria in mice, but when mice fed with high‐fat chow after 5 months significantly increased in albuminuria by 2.5 ± 1.3‐fold when compared to normal chow (Fig. 1C,D, *P* < 0.05).

**Table 1:** General characteristics of the rat experimental groups

	ZL	ZF
Number	6	4
Initial body weight	169 ± 13	217 ± 7*
Body weight at 2 months	303 ± 17	482 ± 10*

ZL, Zucker lean rats; ZF, Zucker fatty rats. Data are expressed as means ± SD. *P < 0.05 versus ZL rats.

**Table 2:** General characteristics of the mice experimental groups

	Normal chow	High fat
Number	6	4
Initial body weight	25 ± 2	25 ± 2
Body weight after 5 months	34 ± 4	46 ± 2*

Data are expressed as means ± SD. *P < 0.05 versus normal chow.

**Table 3:** Serum triglyceride, serum total cholesterol, and plasma insulin

	ZL	ZF
Triglyceride	28.1 ± 14.3	195.7 ± 23.6*
Total cholesterol	51.2 ± 5.9	77.5 ± 7.0*
Insulin	21.9 ± 0.5	19.9 ± 0.5

ZL, Zucker lean rats; ZF, Zucker fatty rats. Data are expressed as means ± SD. *P < 0.05 versus ZL rats.

### Glomerular inflammation in experimental groups

**Figure 2:**
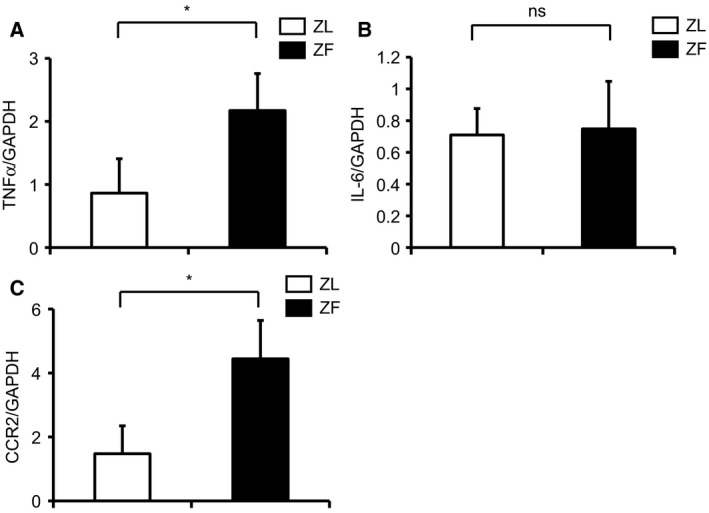
Evaluation of inflammatory markers in the glomeruli of ZL and ZF rats. (A) TNF‐α mRNA expression in the glomeruli of ZL and ZF rats. (B) IL‐6 mRNA expression in the glomeruli of ZL and ZF rats. (C) CCR2 mRNA expression in the glomeruli of ZL and ZF rats. *P < 0.05. ns, not significant. These data are expressed as means ± SD. ZL, Zucker lean rats; ZF, Zucker fatty rats.

Inflammatory markers were characterized in the glomeruli with the induction of obesity. Expression of TNF‐α mRNA and CCR2 mRNA was elevated by 3.3 ± 2.4‐ and 3.1 ± 1.5‐fold in the glomeruli of ZF rats, respectively, when compared with ZL rats (Fig. 2A,B, *P* < 0.05). In contrast, expression of IL‐6 mRNA did not increase in the glomeruli of ZF rats after 2 months of feeding (Fig. 2C), which is consistent with our previous report 11.

### Renal histology in experimental groups

**Figure 3:**
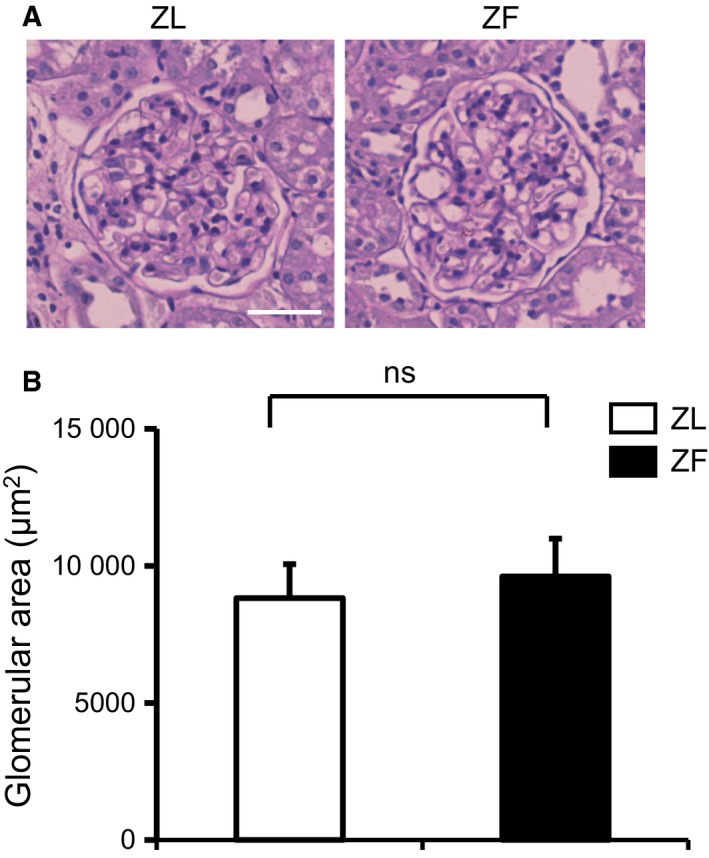
Renal morphology in the experimental groups. (A) Representative light microscopic appearance of glomeruli (periodic acid–Schiff) for ZL and ZF rats. Bar = 50 μm. (B) Morphometric analysis of glomerular area. ns, not significant. These data are expressed as means ± SD. ZL, Zucker lean rats; ZF, Zucker fatty rats.

We next performed morphometric analysis of glomerular surface area. There were no statistically significant differences in the glomerular surface area between ZL and ZF rats (Fig. 3; ZL, 8820 ± 1240 μm^2^; ZF, 9612 ± 1384 μm^2^, respectively).

### Immunohistochemistry of NF‐κB activation in the glomeruli of mice fed a high‐fat diet

**Figure 4:**
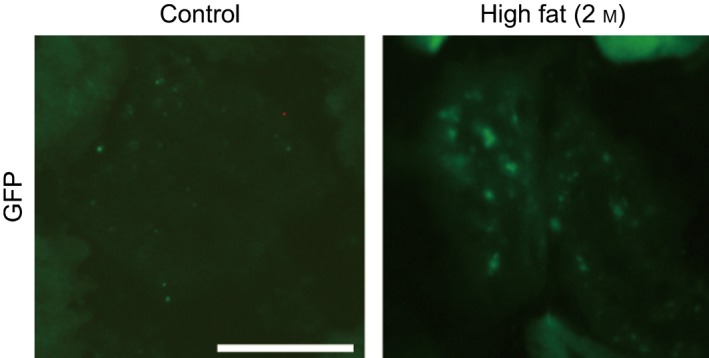
Activation of NF‐κB in the glomeruli of cis‐NF‐κB^EGFP^ transgenic mice. cis‐NF‐κB^EGFP^ transgenic mice were fed a high‐fat diet for 2 months, after which EGFP fluorescence was assessed using digital fluorescence microscopy. Bar = 50 μm.

As inflammation and oxidative stress can activate NF‐κB in obesity and the insulin‐resistant state 12, we examined changes in NF‐κB activity in the glomeruli of cis‐NF‐κB^EGFP^ mice. GFP‐positive areas were detected in the glomeruli of mice fed a high‐fat diet for 2 months, indicating NF‐κB activation (Fig. 4).

## Discussion

This study reports for the first time that obesity and the insulin‐resistant state increase albuminuria, which is correlated with inflammation or oxidative stress. However, 2 months of metabolic abnormality was not enough to cause glomerular pathological changes. Previous reports have primarily focused on the activation of inflammation and oxidative stress by diabetes alone. Several studies have associated changes in inflammation and oxidative stress with albuminuria in CKD 13, 14. However, it has not been reported that inflammation and oxidative stress can increase albuminuria, in the absence of renal pathological changes.

Quantitative PCR data indicated elevation of TNF‐α and CCR2, but not IL‐6 in the glomeruli of obese animals. Previously, we have reported that levels of TNF‐α and IL‐6 mRNA are increased by diabetes, but not by insulin resistance in retina 11. Also, it is reported that obesity‐induced increases in IL‐6 did not correlate with the incidence rate of acute kidney injury, while oxidative stress marker plasma F2‐isoprostanes was increased in those patients 15. Expression of IL‐6 mRNA was mainly recognized in moderate mesangial expansion area and the interstitial expression correlated with the degree of interstitial damages in diabetic kidney disease (DKD) 16. Thus, increases in IL‐6 level could be recognized in the kidney that was damaged to some extent by insulin‐resistant state or diabetes. These findings could support the expression discrepancy between TNF‐α and IL‐6 mRNA in our study.

Our data suggest that obesity could increase TNF‐α and CCR2 in the kidney, when hyperinsulinemia is not present. Recent studies clearly show that TNF‐α and its receptors, TNF receptors 1 and 2, are correlated with estimated glomerular filtration rate 17. Interestingly, these inflammatory markers are significantly increased in CKD patients without diabetes 18.

The mechanism for obesity‐induced proteinuria appears to be via TNF‐α activation. Among the inflammatory cytokines that were activated in the glomeruli, TNF‐α appears to be the primary contributor to increased proteinuria. Previous studies indicated that TNF‐α could change membrane permeability 19 resulting in proteinuria 20. Clinically, inhibition of TNF‐α using the TNF‐α neutralizing antibody, tocilizumab, results in decreased proteinuria.

Some reports have suggested that high‐fat feeding over a prolonged time could develop mesangial expansion 21. However, glomerular histological changes were not recognized in our study. Our observation period was only 2 months, while previous studies that showed high‐fat diet‐induced renal injuries were more than 3 months. Furthermore, they used the diet 60% from fat, while 42% in our study.

It is also possible that monocyte chemoattractant protein (MCP)‐1/CCR2 pathway can be a pivotal role in developing DKD 22, 23. Furthermore, our previous study directly proved CCR2 contributed to the progression of DKD using CCR2 antagonist, propagermanium 24. Recent study using CCR2 inhibitor, CCX140‐B and being excluded advanced nephropathy showed renoprotective effects, reducing albuminuria in DKD patients 25. Our results support the idea that inflammatory cytokines may be elevated before developing renal pathological changes and inhibiting cytokine action as a possible therapeutic target could improve and prevent DKD.

Our previous work suggested that PKC activation selectively inhibits insulin/insulin receptor (IRS)1 signaling, increasing inflammation and oxidative stress in the glomerulus of ZF rats 3. Here, we demonstrate that obesity, without diabetes, induced by a high‐fat diet was able to activate NF‐κB in the glomerulus. In addition, increased TNF‐α in the glomerulus can induce albuminuria after 2 months of obesity in ZF rats. Activation of the tyrosine phosphatase, Src homology‐domain‐containing phosphatase‐1 (SHP‐1), which is increased by diabetes and PKC‐δ, causes VEGF resistance‐induced podocyte apoptosis 26. Mechanistically, this pathway is independent of inflammation, oxidative stress, and NF‐κB.

In summary, obesity can elevate the inflammatory cytokine, TNF‐α and CCR2, resulting in increases in albuminuria. Moreover, obesity‐activated NF‐κB is correlated with inflammation and oxidative stress in the glomerulus. However, 2 months of disease duration may not be long enough to develop renal pathological changes. Further understanding of the NF‐κB, TNF‐α, and CCR2 pathways could lead to effective interventions for obesity‐induced CKD.

## Author contributions

AM and TY researched the data. AM wrote the manuscript, researched the data, reviewed, and edited the manuscript. GLK and SU contributed to the discussion. SU reviewed and edited the manuscript. AM and TY contributed equally to this work.
